# Novel use of a chemically modified siRNA for robust and sustainable in vivo gene silencing in the retina

**DOI:** 10.1038/s41598-020-79242-w

**Published:** 2020-12-18

**Authors:** Takazumi Taniguchi, Ken-ichi Endo, Hidetoshi Tanioka, Masaaki Sasaoka, Kei Tashiro, Shigeru Kinoshita, Masaaki Kageyama

**Affiliations:** 1 Research and Development Division, Santen Pharmaceutical Co., Ltd., 8916-16 Takayama-cho, Ikoma-shi, Nara, 630-0101 Japan; 2 Global Alliances and External Research, Santen Pharmaceutical Co., Ltd, Nara, Japan; 3 Department of Genomic Medical Sciences, Kyoto Prefectural University of Medicine, Kyoto, Japan; 4 Department of Frontier Medical Science and Technology for Ophthalmology, Kyoto Prefectural University of Medicine, Kyoto, Japan

**Keywords:** Retinal diseases, Target identification

## Abstract

Despite efficient and specific in vitro knockdown, more reliable and convenient methods for in vivo knockdown of target genes remain to be developed particularly for retinal research. Using commercially available and chemically modified siRNA so-called Accell siRNA, we established a novel in vivo gene silencing approach in the rat retina. siRNA designed for knockdown of the house keeping gene *Gapdh* or four retinal cell type-specific genes (*Nefl*, *Pvalb*, *Rho* and *Opn1sw*) was injected into the vitreous body, and their retinal mRNA levels were quantified using real-time PCR. Intravitreal injection of siRNA for *Gapdh* resulted in approximately 40–70% reduction in its retinal mRNA levels, which lasted throughout a 9-day study period. Furthermore, all the selected retinal specific genes were efficiently down-regulated by 60–90% following intravitreal injection, suggesting injected siRNA penetrated into major retinal cell types. These findings were consistent with uniform distribution of a fluorescence-labeled siRNA injected into the vitreous body. Interestingly, gene silencing of *Grin1*, a core subunit of NMDA receptor, was accompanied by significant prevention from NMDA-induced retinal ganglion cell death. Thus, we provide single intravitreal injection of Accell siRNA as a versatile technique for robust and sustainable in vivo retinal gene silencing to characterize their biological functions under physiological and pathophysiological conditions.

## Introduction

Gene silencing by small interfering RNA (siRNA) has been an indispensable tool to analyze biological functions of genes of interest. More importantly, this strategy may have potential as an alternative therapy for human diseases that cannot be managed by conventional therapeutic modalities^1,2^. In fact, the therapeutic potentials of siRNA-related drugs for ocular diseases, namely, bevasiranib, AGN211745 and PF-04523655 for age-related macular degeneration (AMD), and SYL040012 and QPI-1007 for glaucoma, have been assessed in clinical trials, which provide promising results for some of those drugs^3^. siRNA-mediated gene silencing has also been used for retinal research, resulting in identification of several genes responsible for retinal damage in in vivo animal disease models such as ischemia, optic nerve transection, and diabetes^4–8^. In most cases, a siRNA is injected into the vitreous body, often combined with a transfection reagent to promote intracellular delivery of the siRNA^8–10^. Despite their methodological benefits, transfection reagents may have the potential for non-specific retinal toxicity, and eliciting reactive immune responses^1,2^. Two alternative methods, a viral vector and electroporation of a plasmid DNA, have commonly been used to deliver siRNA into the retina^11–13^. However, these delivery methods require special skills for successful implementation of what may be cumbersome procedures. For these reasons, a simpler and more reliable gene silencing technique using siRNA needs to be developed to further accelerate in vivo functional analysis of retinal genes.

Recently, the use of chemical modification of siRNA has evolved to dramatically improve the stability, efficiency, and specificity of siRNA-mediated gene silencing. Dharmacon has developed a novel Accell siRNA technology that includes a proprietary sequence design algorithm to reduce off-target effects^14^ and unique chemical modifications of siRNA molecular structures. This technology does not require any transfection reagents and can be applied to transfection-resistant cell types including in vitro primary neuronal cells^15,16^. Interestingly, an Accell siRNA was used for in vivo studies with successful achievement of gene silencing in multiple organs and tissues^17–20^. In particular, Accell siRNA-mediated in vivo gene silencing in the brain suggests that this technology also would be useful for the retina^17^.

The present study was designed to investigate the potential of Accell siRNA in gene silencing in vivo in the rat retina. This study comprised three components: First, we quantitatively determined the silencing efficiency for individually targeted genes expressed in specific retinal cell types following a single intravitreal injection of Accell siRNA. Second, we histologically analyzed tissue distribution of a fluorescence-labelled Accell siRNA across the retina. Finally, we determined if gene silencing by an Accell siRNA targeting *Grin1* (a N-methyl-d-aspartate or NMDA receptor subtype) would protect retinal ganglion cells (RGCs) against NMDA-induced retinal degeneration. Our results provide evidence for robust and sustainable in vivo silencing of retina-specific genes by a simple and reliable technique, using an Accell siRNA in rats.

## Results

### Quantitative assessment of efficiency of gene silencing by a single intravitreal injection of Accell siRNAs

**Figure 1:**
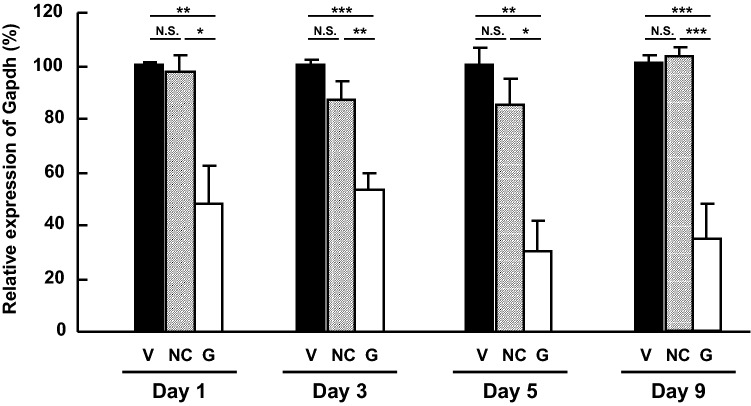
Time-gene silencing profiles following intravitreal injection of Accell siRNA targeting *Gapdh* in the rat retina. Gene expression of *Gapdh* was measured by means of quantitative real-time PCR at 1, 3, 5 and 9 days after intravitreal injection of vehicle, negative control siRNA (2 nmol/eye for Day 1 and 5 nmol/eye for Day 3, 5 and 9), or siRNA (2 nmol/eye) targeting *Gapdh*. Gene expression of *Gapdh* was normalized to that of *Actb* and is shown as the percentage of the respective control value in the vehicle-treated group. Each value represents the mean ± S.E.M. for n = 3–6. *P < 0.05; **P < 0.01; ***P < 0.001, by Tukey’s multiple comparison test. N.S. represents not significant. Vehicle (V), closed column; negative control siRNA (NC), gray column; *Gapdh* siRNA (G), open column.

To assess gene silencing efficiency of Accell siRNA and optimize the experimental conditions in vivo, we first determined mRNA levels of a housekeeping gene following intravitreal injection of siRNA, targeting *Gapdh* by real-time PCR. Figure 1 shows the time course of changes in mRNA levels of *Gapdh* gene in the rat retinas treated with specific siRNA, relative to those observed in the vehicle (Dulbecco's phosphate-buffered saline or D-PBS only)-treated control retinas. At Day 1 following injection of 2 nmol/eye negative control (NC) siRNA, the *Gapdh* mRNA levels were not significantly different (*P* = 0.9778) from the levels observed with vehicle injection. Even when the dose of NC siRNA was increased to 5 nmol/eye, it did not alter *Gapdh* gene expression determined at Day 3, 5 and 9 post-injection, compared with the vehicle injection (*P* = 0.3070 at Day 3, *P* = 0.5955 at Day 5 and *P* = 0.9846 at Day 9). In contrast, injection of 2 nmol/eye siRNA targeting *Gapdh* statistically significantly down-regulated gene expression by 51% at Day 1 following siRNA injection, when comparing with NC (*P* = 0.0103). When *Gapdh* gene expression was measured at Day 3 through Day 9 following injection, *Gapdh* specific siRNA reduced it by 39–67% in statistically significant fashion at all time points, compared with NC (*P* = 0.0081 at Day 3, *P* = 0.0100 at Day 5 and *P* = 0.0003 at Day 9). Similar results were also observed when comparing with the vehicle. The magnitude of this reduced gene expression was consistent throughout the 9-day study period. These results indicate that a single intravitreal injection of Accell siRNA produced efficient and sustainable in vivo gene silencing for up to 9 days and that the dose of siRNA can be increased up to 5 nmol/eye without causing non-specific knockdown of a targeted gene.

**Figure 2:**
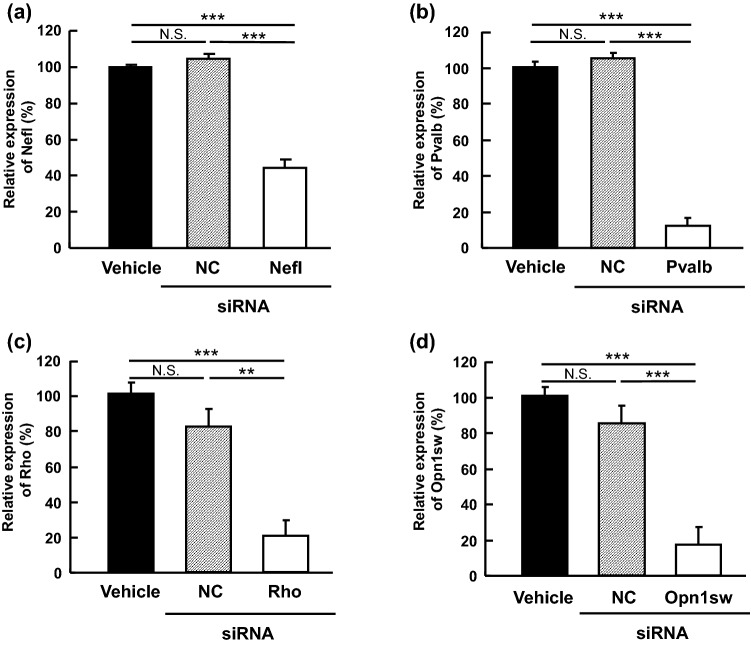
Gene silencing following intravitreal injection of Accell siRNA targeting four retinal cell type-specific genes in the rat retina. Expression of cell type-specific genes was measured 3 (*Pvalb*, *Rho* and *Opn1sw*) or 4 days (*Nefl*) after intravitreal injection of vehicle (closed column) or negative control (4 nmol/eye, NC, gray column), or siRNAs targeting cell type-specific genes (4 nmol/eye, open column), as determined by quantitative real-time PCR. The expression values of cell type-specific genes were normalized to that of *Gapdh* [*Nefl*, (**a**); *Pvalb*, (**b**); *Opn1sw*, (**d**) or *Actb* (*Rho*, **(c**))] and is shown as the percentage of the respective control value in vehicle-treated group. Each value represents the mean ± S.E.M. for n = 4 or 8. **P < 0.01; ***P < 0.001, by Tukey’s multiple comparison test. N.S. represents not significant.

Next, we assessed gene silencing by Accell siRNAs directed at target genes specifically expressed in each of four retinal cell types: The selected genes were *Nefl*, *Pvalb*, *Rho* and *Opn1sw*, which are specifically expressed in RGC, subtype AII amacrine cells, and rod and cone photoreceptor cells, respectively. As shown in Fig. 2a–d, gene expression, as determined for total RNA isolated from the whole retina, was noticeably down-regulated 3 (*Pvalb*, *Rho* and *Opn1sw*) or 4 days (*Nefl*) following single intravitreal injections of siRNA (4 nmol/eye) targeting each cell type-specific gene. The reductions in gene expression ranged from 58 to 89% of NC values across all four genes, and these results were all statistically significant versus the respective NC siRNA (*P* = 0.0013 in *Rho*, *P* = 0.0009 in *Opn1sw*, *P* < 0.0001 in *Nefl* and *Pvalb*). Similar results were also observed when comparing to the vehicle group. The NC siRNAs did not statistically affect the gene expression of any of the targeted genes in comparison with the vehicle control group (*P* = 0.5468 in *Nefl*, *P* = 0.6043 in *Pvalb*, *P* = 0.3125 in *Rho* and *P* = 0.4276 in *Opn1sw*). These results indicate that single intravitreal injections of siRNA are sufficient to down-regulate genes specifically expressed in these selected retinal cell types.

### Histological analysis of retinal localization of siRNA following an intravitreal injection of a fluorescence-labelled Accell siRNA

**Figure 3:**
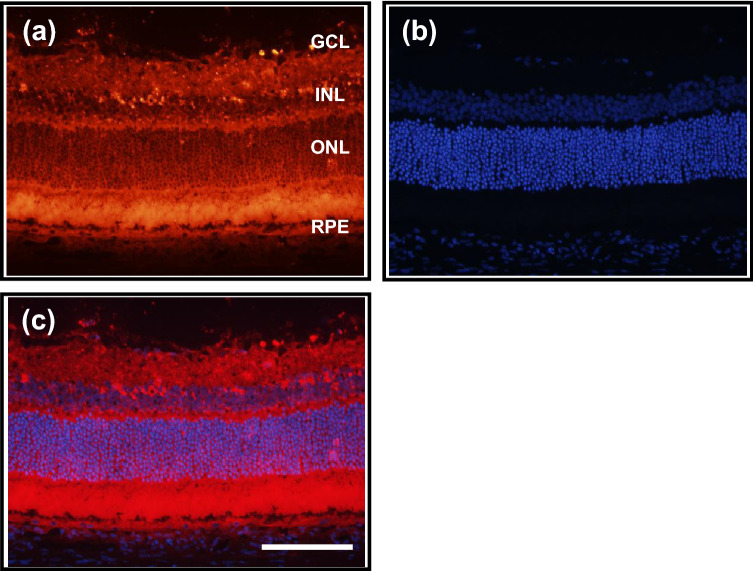
Spatial distribution of fluorescence-labeled Accell siRNA following intravitreal injection in the rat retina. Histological analysis of retinal tissues was performed 3 days after intravitreal injection of fluorescence-labeled negative control siRNA (2 nmol/eye). (**a**) siRNA; (**b**) DAPI staining; (**c**) Merge of siRNA and DAPI images. The scale bar represents 100 μm. *GCL* ganglion cell layer, *INL* inner nuclear layer, *ONL* outer nuclear layer, *RPE* retinal pigment epithelium.

The quantitative measurements of gene silencing in the whole retina presented above suggests a uniform distribution of siRNA across the retina. To determine more precise retinal localization of siRNA, a fluorescence-labeled Accell siRNA (2 nmol/eye) was injected into the vitreous body and its retinal localization was histologically determined (Fig. 3a–c). Three days following a single intravitreal injection of a labelled siRNA, intense fluorescence was observed across the retina from the ganglion cell layer up to the retinal pigment epithelium (RPE). Interestingly, the detected signal was strong in the inner plexiform layer, the outer plexiform layer, the photoreceptor layer and RPE, whereas it was weak in the inner nuclear layer and the outer nuclear layer. These fluorescence signals were not observed in the eye in which only D-PBS (vehicle) was injected, under the microscope condition used in evaluation of above labelled siRNA (Supplementary Fig. S1). An overlay image of the retina dual-labeled with a fluorescence-conjugated siRNA and DAPI indicates that siRNA is predominantly localized in the cytosol of each retinal layer. As shown in Supplementary Fig. S2, no signs of abnormal structural changes or cellular infiltration caused by inflammation were observed either in the retina or the vitreous 7 days after the injection of 7.0 nmol/eye NC siRNA, which was higher than the effective doses for knockdown of the targeted genes (2 and 4 nmol/eye). Thus, the exclusively cytosolic localization of siRNA following intravitreal administration supports the results of quantitative assessment of gene silencing. This is also consistent with the functional roles and the mode of action of siRNA, namely, being incorporated into the RNA-induced silencing complex (RISC) in the cytosol and interfering with the translation of target mRNA.

### Inhibition by Accell siRNA targeting Grin1 of retinal damage induced by NMDA following a single intravitreal injection

**Figure 4:**
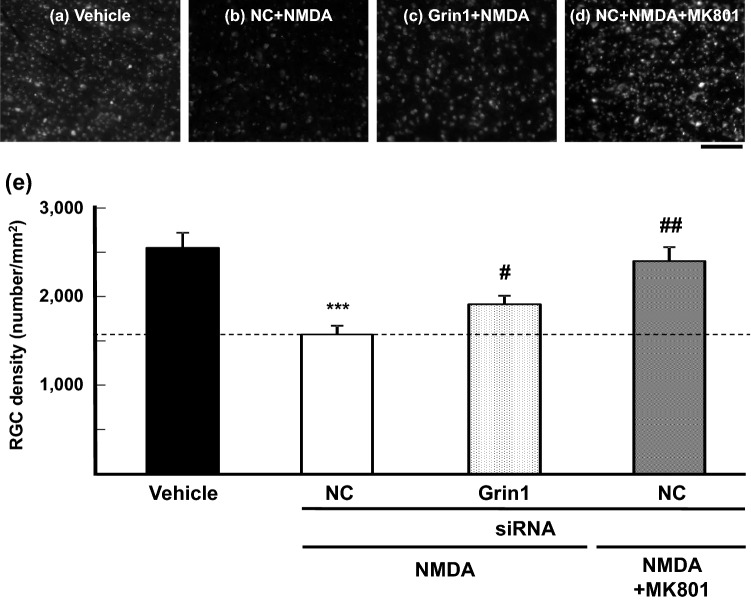
Inhibition of NMDA induced-retinal ganglion cell death following intravitreal injection of siRNA targeting *Grin1. *The photographs represent fluorogold-labeled RGCs in the flat-mounted rat retina treated with vehicle (**a**), negative control siRNA (NC) + NMDA (**b**), *Grin1* siRNA + NMDA (**c**) and NC + NMDA + MK-801 (**d**) 7 days after intravitreal injection of NMDA. The scale bar represents 100 µm. The number of fluorogold-labeled RGCs was quantified on each image and is shown in (**e**). Vehicle, closed column; NC + NMDA, open column; *Grin1* siRNA + NMDA, light gray column; NC + NMDA + MK-801, dark gray column. Each value represents the mean ± S.E.M. for n = 4–8. ***P < 0.001 versus vehicle (Student’s *t*-test), ^#^*P* < 0.05, ^##^*P* < 0.01 versus NC siRNA with NMDA (Student’s *t*-test).

To determine if Accell siRNA might substantially modulate target protein functions in the retina via reduced mRNA and protein levels, we assessed the effects of a single intravitreal injection of Accell siRNA on NMDA induced-retinal damage. This rat model is often used to characterize the roles for glutamate receptors, namely the NMDA receptor subtype, in ocular diseases, including glaucoma, that incorporate RGC death^21–23^. As expected, intravitreal injection of NC siRNA in combination with NMDA (10 nmol/eye, Fig. 4b,e) resulted in a statistically significant decrease in the number of RGCs (*P* = 0.0004), compared with application of vehicle alone (Fig. 4a,e). In contrast, intravitreal injection of Accell siRNA targeting *Grin1*, a core subunit of the NMDA receptor expressed predominantly in RGCs^24^, reduced RGC death by 35%, in a statistically significant fashion (*P* = 0.0294), when combined with NMDA (Fig. 4c,e). This trend mimics the inclusion of MK-801, an authentic NMDA receptor antagonist, which remarkably prevented NMDA-induced RGC death (Fig. 4d,e). In separate experiment, the same siRNA targeting *Grin1* reduced gene expression of *Grin1* by approximately 45%, compared with NC (relative expressions of *Grin1* in NC and *Grin1* siRNA treatment group were 104.3 ± 8.4 (n = 4) and 57.5 ± 23.9 (n = 3), respectively). These results suggest that decreased mRNA and possibly protein levels of *Grin1* by Accell siRNA resulted in reduced NMDA receptor activation, as also demonstrated with a receptor antagonist.

## Discussion

We demonstrate here the development of a novel and simple technique using Accell siRNA for reliable retinal gene silencing in vivo. The key finding of the current study is that a single injection of Accell siRNA into the adult rat vitreous body results in distribution of the agent to the entire retina, thereby promoting consistent and sustainable silencing of targeted genes. Gene silencing efficiency by Accell siRNA varied in the range of 39–89% depending on targeted genes. However, the magnitude of gene silencing was consistent throughout the study periods involved, and was maintained for at least 9 days in case of *Gapdh*. More importantly, intravitreal administration of Accell siRNA directed against *Grin1* was sufficient to induce at least partial protection against retinal damage caused by NMDA receptor stimulation, similar to those seen with NMDA receptor antagonism. Interestingly, gene silencing by Accell siRNA is not limited to genes expressed in RGCs, which are anatomically close to the injection site; following intravitreal injection, siRNAs were shown to be uniformly distributed across the retina, even reaching beyond the original injection site to the photoreceptor layer, presumably the specific site of action where two photoreceptor specific genes were equivalently down-regulated. These results substantiate robust gene silencing by Accell siRNA in any of the retinal specific genes following intravitreal injection.

Accell siRNA was originally discovered and developed by Dharmacon, which has extensively characterized its gene silencing ability. According to the manufacturer, its proprietary algorithm enables to predesign and optimize a specific siRNA for any genes of interest with minimal off-target events as assessed by genome-wide gene expression profiling^14,25^. They also claim that these designed siRNAs do not induce an innate immune response, including TLR3-mediated cytokine production, which may be a potential confounding factor for data interpretation. This statement is substantiated by the finding that IL-6 and IL-8 release as the key mediators of the TLR3 immune response was not altered in the cultured HeLa S3 cells^25,26^. In this study, we also observed that NC siRNA had no effect on the expression of any of the selected genes, whereas gene-specific siRNAs significantly down-regulated that of the respective targeted gene, suggesting minimal off-target effects of Accell siRNAs on the expression of the selected genes. Furthermore, we found no signs of intraocular inflammation following NC siRNA injection: the anatomical structures of the retina and the vitreous were well-preserved and neither of them was infiltrated with any cells. As such, we believe that in vivo retinal gene silencing induced by the selected gene-specific Accell siRNAs are not confounded by their potential off target effects or nonspecific immune responses via the TLR3 pathway, if any.

In vivo gene silencing efficiency in this study was somewhat reduced, to only approximately 40–80% in most cases and 90% at maximum compared with in vitro efficiency. However, this magnitude of gene silencing was comparable to or even higher than that achieved in many earlier in vivo studies^5,8,9,27,28^. The variations in the extent of gene silencing we obtained most likely are not due to skewed distribution of injected siRNA in the retina, because we observed its uniform distribution across the retina in both cross-sectional dimensions. Although the exact mechanisms underlying the observed range of gene silencing are unclear, differences in siRNA sequence designs and optimal concentrations among all the tested genes may partly play a role. Further optimization of experimental conditions for Accell siRNA is necessary for better gene silencing efficiency for each targeted gene.

In earlier studies, unmodified and naked siRNA was simply dissolved in saline and administered into the eye^6,29^. Although gene silencing efficiency was not quantitatively determined in some studies, relatively moderate knockdown of targeted genes was observed with this simple procedure. To improve gene silencing efficiency, transfection reagents were often used^8–10^. However, the use of transfection reagents may have the potential to induce associated retinal toxicity or inflammatory responses^1,2^. Both a viral vector encoding siRNA as well as electroporation of siRNA-expressing plasmid would appear to be ideal methods to obtain robust gene silencing^11,12,30^. A drawback of these methods is that they require vector construction, setting up and optimizing equipment, and subretinal injection, all of which contribute to added elements of complexity and surgical invasiveness. In contrast, our Accell siRNA-based in vivo gene silencing technique is very simple and easy: predesigned and optimized Accell siRNAs can be obtained for targeted genes of interest, and readily dissolved in D-PBS and injected into the vitreous body. As demonstrated here, siRNA can be efficiently delivered to the retina with a single intravitreal injection of siRNA, sufficient for robust gene silencing even in the absence of a transfection reagent. In this way, our in vivo gene silencing technique provides an alternative and versatile approach for any investigations of functional analysis of retinal genes in vivo.

Other unique features of our technique are uniform retinal distribution of siRNA and sustainable gene silencing following single intravitreal injection. When fluorescently-labelled Accell siRNA was injected into the vitreous body, it rapidly diffused, penetrated and uniformly distributed throughout the retina. siRNA even reached the RPE layer which is most distant from the injection site in the vitreous body. Efficient gene silencing for the four selected genes indicate that Accell siRNA was present and active in RGC, amacrine, and both general classes of photoreceptor cells. The uniform subcellular distribution pattern of Accell siRNA in the retina, with the exception of the nuclear compartments, also suggests that gene silencing can be accomplished in other retinal cell types, namely, bipolar, horizontal, Müller cells, and RPE. Furthermore, the observed duration of gene silencing by Accell siRNA is expected to be sufficient to analyze functional roles for short half-life proteins in acute animal disease models. For proteins with longer-half lives and chronic animal models, repeated intravitreal injection of Accell siRNA is possible. Given that intravitreal injection is routinely performed even in mice and that Accell siRNA is available for mouse genes, this technique can be complementary to genetic engineering, such as the use of knockout mice to analyze functional roles for targeted genes in the rodent retina in vivo.

Importantly, our study demonstrated that gene silencing by siRNA targeting *Grin1* led to significant protection of RGCs against NMDA-induced retinal damage. GRIN1 is a core subunit of the NMDA receptor, which triggers calcium influx in response to its binding by glutamate, and eventually activates a neuronal cell death pathway^31^. Many studies have reported that intravitreal injection of NMDA causes massive degeneration especially in the inner retina including RGCs, and this effect is completely inhibited by concomitant injection with MK-801, a non-competitive NMDA receptor antagonist, in rats^22,23^. These earlier findings are consistent with this study, providing evidence that Accell siRNA-mediated gene silencing can modify functional activities of targeted gene products. In preliminary experiments studying gene silencing efficiency for *Grin1* in a small number of animals, we found that gene expression of *Grin1* was reduced by approximately 45%, compared with NC. The degree of knockdown of *Grin1* was similar to the measured 35% inhibition by siRNA of NMDA-induced RGC death. One possible interpretation of our results is that the levels of functional gene and protein expression of, and the resultant physiological activity associated with, the NMDA receptor complex in the retina may be quantitatively correlated following siRNA treatment. This further supports the use of our in vivo gene silencing technique to determine functional roles for genes/proteins in the retina and improve throughput levels of drug target discovery in rodent retinal disease models.

In summary, we developed a simple and reliable technique for robust in vivo retinal gene silencing in rats. This technique consists of an intravitreal injection of Accell siRNA, a chemically modified siRNA, which does not require any transfection reagents or any cumbersome procedures including specialized devices or equipment. Gene silencing is rapid and sustainable throughout the study. With an appropriate design and optimization of siRNA, gene silencing can be achieved to modulate sufficiently the functional activities of gene products in any retinal cell across all the retinal layers. Given such unique features, our technique may be an alternative tool to complement other techniques, including genetic engineering and viral gene delivery to characterize physiological and pathophysiological roles for genes/proteins in animal disease models. More extensive studies would be required to determine if Accell siRNA-based gene silencing may have a potential to be an alternative medical therapy for human retinal diseases which cannot be addressed with the therapeutic modalities currently in use.

## Methods

### Preparation of siRNA

Accell siRNA was purchased from Thermo Scientific (MA, USA). Lyophilized siRNA was reconstituted in D-PBS. The concentration of dissolved siRNA was measured using an ultraviolet (UV) wavelength range spectrophotometer and was adjusted to final concentrations of 0.4, 0.8, 1 or 1.4 mM for intravitreal injection (5 μL/eye) according to each experimental condition (see below). Product codes of siRNAs with target transcripts are as follows: D-001910-10 for negative control, D-001960-01 for fluorescence-labeled negative control, D-001930-03 for *Gapdh* (NM_017008), E-096955 for *Nefl* (NM_031783), EU-091951 for *Rho* (NM_033441), E-088578 for *Pvalb* (NM_022499), E-093092 for *Opn1sw* (NM_031015) and EU-080174 for *Grin1* (NM_017010).

### Animals

Male Sprague–Dawley rats weighing 105.8–151.4 g (Charles River Laboratories Japan, Inc., Yokohama, Japan) were maintained under controlled light/dark conditions, with food and water available ad libitum. All animal care and experimental procedures were performed in accordance with the ARVO Statement for the Use of Animals in Ophthalmic and Vision Research and were approved and monitored by the Animal Care and Use Committee at Santen Pharmaceutical Co., Ltd.

### Injection of siRNA into retina

siRNA was injected intravitreally as described in our previously published study, with minor modifications^22,32–34^. Briefly, siRNA solution (5 μL/eye, for 2, 4, 5 or 7.0 nmol/eye) was injected into the vitreous body of eye of anesthetized animals with 1.5% isoflurane. Intravitreal injections were accomplished by careful insertion of a 32-gauge needle attached to a Hamilton syringe, with the aid of a surgical microscope. As a vehicle control for siRNA injection, an equal volume of D-PBS was injected intravitreally in the same manner.

### Isolation of RNA from retina and reverse transcription

At 1, 3, 4, 5 or 9 days after the injection of vehicle or siRNA into the vitreous body, eyeballs were enucleated from euthanized animals. Individual retinas were then carefully isolated, immersed in RNAlater (Ambion, TX, USA), and stored at 4 °C until subsequent lysis, which was carried out in 450 μL of Qiazol lysis reagent (Qiagen, Hilden, Germany) for 5 min using a TissueLyser (Qiagen) at a setting of 25 Hz. After mixing each sample lysate with 90 μL of chloroform, 200 μL of the resulting aqueous layer was isolated following centrifugation. After addition of 200 μL 70% ethanol, samples were transferred to an RNeasy 96 plate (Qiagen), and total RNA isolation was performed using the RNeasy 96 kit (Qiagen) according to the manufacturer’s instructions. The amount and quality of the isolated RNA were evaluated by UV spectrophotometry. After dilution of RNA with RNase-free water, volumes corresponding to 200 ng RNA were immediately used for reverse transcription to produce cDNA, by means of a PrimeScript RT reagent Kit (Takara, Shiga, Japan), in accordance with the manufacturer’s instructions.

### Quantitative real-time PCR

To quantify the expression of genes of interest other than *Nefl*, target gene-specific primers (Table 1), purchased from Takara were used with the QuantiFast™ SYBR^Ⓡ^Green PCR Kit (Qiagen) according to the manufacturer’s instructions. Briefly, cDNA was amplified in the presence of SYBR Green PCR Master Mix in a final reaction volume of 20 μL per well using the 7500 Fast Real-Time PCR System (Life Technologies, CA, USA). *Nefl* transcript was amplified with *Gapdh* transcript by using a specific TaqMan probe (Table 1) with a QuantiTect Multiplex PCR Kit (Qiagen) according to manufacturer’s instructions. Primers and probe for *Nefl* were designed by Oligo@SIGMA-DLP (Sigma-Aldrich, Saint Louis, MO, USA), while *Gapdh* primers and probe were designed by TaqMan Gene Expression Assays (Life Technologies).

**Table 1:** Transcript and primer information for quantitative real-time PCR.

Gene symbol	Full name of gene	Accession no. of transcript	Strand	Primer sequence (5′–3′)
*Gapdh*	Glyceraldehyde-3-phosphate dehydrogenase	NM_017008	F	GACAACTTTGGCATCGTGGA
			R	ATGCAGGGATGATGTTCTGG
*Rho*	Rhodopsin	NM_033441	F	ACACCTCACTGCATGGCTACTTTG
			R	TTGCTCATGGGCTTGCAGAC
*Pvalb*	Parvalbumin	NM_022499	F	TGTTCCACATTCTGGACAAAGACAA
			R	AGCAGACAAGTCTCTGGCATCTGA
*Opn1sw*	Opsin 1 (cone pigments), short-wave-sensitive	NM_031015	F	GCTGTACTTACGGCTTGTCACCA
			R	CACACCATCTCCAGGATGCAG
*Actb*	Actin, beta	NM_031144	F	GGAGATTACTGCCCTGGCTCCTA
			R	GACTCATCGTACTCCTGCTTGCTG
*Gapdh*	Glyceraldehyde-3-phosphate dehydrogenase	NM_017008	F	Nondisclosure
			R	Nondisclosure
			Probe	VIC-nondisclosure-MGB
*Nefl*	Neurofilament, light polypeptide	NM_031783	F	ACAAGCAGAATGCAGACATCA
			R	GGAGGTCCTGGTACTCCTTC
			Probe	FAM-CCATCTCGCTCTTCGTGCTTCGC-BHQ-1

*F* forward, *R* reverse.

PCR reactions were analyzed by using 7500 Fast System SDS Software Version1.4 software (Applied Biosystems, CA, USA). Each gene expression was determined by the standard ΔΔCt method. Duplicate cycle threshold (Ct) values were obtained from each retinal sample, and their average was used for further calculations. The ΔCt was calculated by normalizing against each reference gene. As reference genes, we used β-actin (*Actb*) for *Gapdh* and *Rho*, and *Gapdh* for *Nefl*, *Pvalb* and *Opn1sw*. The ΔCt was then compared to the expression levels of the vehicle-treated group and ΔΔCt was calculated. The relative expression of each gene of interest was calculated using the formula: fold change = 2^−ΔΔCt^.

### Histological analysis of retinal tissue

Three days after the intravitreous injection of fluorescence-labeled NC siRNA, eyeballs enucleated as described above were frozen in OCT compound (Sakura Finetechnical, Tokyo, Japan) using liquid nitrogen. Frozen sections (5 µm thick) cut through the optic disc of each eye were prepared by cryostat (MC3000, LEICA, Tokyo, Japan) and affixed to MAS-GP adhesion slides (Matsunami Glass, Osaka, Japan). Sections were stained with DAPI (Molecular Probes, Thermo Fisher Scientific) and mounted with FLUORO SHIELD (Immuno Bioscience Corp, Mukilteo, US). The distribution of fluorescence-labeled siRNA, as well as nuclear staining by DAPI, was observed using a fluorescence microscope (BX50, OLYMPUS, Tokyo, Japan). Additionally, the retinas exposed to NC siRNA were cross-sectioned, stained with hematoxylin and eosin, and subjected to conventional histological analysis, according to the method reported in our previous studies^22,32,33^.

### Induction of RGC death

To assess the effect of siRNA targeting *Grin1* against RGC death induced by NMDA, RGCs were first retrogradely labeled using the neuronal tracer FluoroGold (FG) as previously described^34^. Four days after the administration of FG, siRNA (4 nmol/eye) was injected intravitreally as described above. Three days after the siRNA injection, NMDA (Sigma-Aldrich; 5 µL/eye, at 10 nmol/eye), to promote RGC death, or D-PBS (vehicle control) was injected into the vitreous bodies of eyes^35^. In a parallel experiment, MK-801 (Sigma-Aldrich) at 10 nmol/eye, was co-injected with NMDA. These intravitreal injections were performed in the same manner as the siRNA injection, described above. Seven days after NMDA injection, eyes were enucleated, and then flat-mounts of retinas were prepared to assay RGC viability (described below).

As in our previous report with minor modifications^34^, images of FG-labeled RGCs in the retina were taken using a SPOT digital camera (Diagnostic Instruments Inc., MI, USA) attached to a fluorescence microscope (Olympus Optical Co. Ltd., Tokyo, Japan) fitted with a 400/475 nm wavelength filter. Each area documented visually in the superior, inferior, nasal and temporal regions was defined as a square of 0.16 mm^2^ area, centered rectilinearly 2 mm from the optic nerve head. To measure RGC density in these areas, we analyzed the images by automation using Image-Pro plus software (Media Cybernetics, Inc., MD, USA). Image analysis was performed using the optimized version [background flattening (width 20), minimum detectable cell size, 50; smoothing intensity, 7; using one pass of the watershed split morphological filter; no use of the large spectral filter and the median enhancement filter] of a program reported in a previous study^36^ to fit with our image analysis. Mean RGC numbers obtained with this program correlated strongly with results obtained using manual methodology, thereby validating the program described above. The data from each area (four regions) were averaged for each eye.

### Statistical analysis

All data are expressed as mean ± S.E.M. For comparison between two groups, statistical analysis was performed using *F*-test, followed by Student’s *t*-test or Aspin-Welch test using EXSAS (Version 7.1.6; Arm, Osaka, Japan). When three groups were compared, one-way analysis of variance (ANOVA) was used and followed by Tukey's multiple comparison test using EXSUS (Version 10.0.3, CAC Croit, Osaka, Japan). *P* < 0.05 was considered statistically significant.

## Supplementary information

Supplementary Information.

## Data Availability

All data generated or analyzed during this study are included in this published article (and its Supplementary Information files).

## Abbreviation

siRNA: Small interfering RNA

## References

[1] Watts JK, Corey DR (2012). Silencing disease genes in the laboratory and the clinic. J. Pathol..

[2] Chen SH, Zhaori G (2011). Potential clinical applications of siRNA technique: benefits and limitations. Eur. J. Clin. Investig..

[3] Jiang J, Zhang X, Tang Y, Li S, Chen J (2020). Progress on ocular siRNA gene-silencing therapy and drug delivery systems. Fundam. Clin. Pharmacol..

[4] Koeberle PD, Wang Y, Schlichter LC (2010). Kv1.1 and Kv1.3 channels contribute to the degeneration of retinal ganglion cells after optic nerve transection in vivo. Cell Death Differ..

[5] Chen J (2009). Suppression of retinal neovascularization by erythropoietin siRNA in a mouse model of proliferative retinopathy. Investig. Ophthalmol. Vis. Sci..

[6] Takeda H (2007). Calcium/calmodulin-dependent protein kinase II regulates the phosphorylation of CREB in NMDA-induced retinal neurotoxicity. Brain Res..

[7] Oku H (2019). Tau is involved in death of retinal ganglion cells of rats from optic nerve crush. Investig. Ophthalmol. Vis. Sci..

[8] Wang J (2006). Nuclear factor-κB p65 and upregulation of interleukin-6 in retinal ischemia/reperfusion injury in rats. Brain Res..

[9] Kaur H (2006). Diabetes-induced extracellular matrix protein expression is mediated by transcription coactivator p300. Diabetes.

[10] Natoli R (2017). Retinal macrophages synthesize C3 and activate complement in AMD and in models of focal retinal degeneration. Investig. Ophthalmol. Vis. Sci..

[11] Michel U, Malik I, Ebert S, Bähr M, Kügler S (2005). Long-term in vivo and in vitro AAV-2-mediated RNA interference in rat retinal ganglion cells and cultured primary neurons. Biochem. Biophys. Res. Commun..

[12] Matsuda T, Cepko CL (2004). Electroporation and RNA interference in the rodent retina in vivo and in vitro. Proc. Natl. Acad. Sci..

[13] Zhao L (2017). Photoreceptor protection via blockade of BET epigenetic readers in a murine model of inherited retinal degeneration. J. Neuroinflamm..

[14] Reynolds A (2004). Rational siRNA design for RNA interference. Nat. Biotechnol..

[15] Vagnoni A (2012). Calsyntenin-1 mediates axonal transport of the amyloid precursor protein and regulates aβ production. Hum. Mol. Genet..

[16] Sebeo J (2009). Requirement for protein synthesis at developing synapses. J. Neurosci..

[17] Nakajima H (2012). A rapid, targeted, neuron-selective, in vivo knockdown following a single intracerebroventricular injection of a novel chemically modified siRNA in the adult rat brain. J. Biotechnol..

[18] Bonifazi P (2010). Intranasally delivered siRNA targeting PI3K/Akt/mTOR inflammatory pathways protects from aspergillosis. Mucosal Immunol..

[19] Gonzalez-Gonzalez E (2010). Silencing of reporter gene expression in skin using siRNAs and expression of plasmid DNA delivered by a soluble protrusion array device (PAD). Mol. Ther..

[20] Cheng C (2012). Endothelial cell-specific fgd5 involvement in vascular pruning defines neovessel fate in mice. Circulation.

[21] Sakamoto K (2017). Activation inhibitors of nuclear factor kappa B protect neurons against the NMDA-induced damage in the rat retina. J. Pharmacol. Sci..

[22] Sasaoka M, Ota T, Kageyama M (2020). Rotenone-induced inner retinal degeneration via presynaptic activation of voltage-dependent sodium and L-type calcium channels in rats. Sci. Rep..

[23] Lam TT, Abler AS, Kwong JMK, Tso MOM (1999). N-Methyl-D-aspartate (NMDA)–induced apoptosis in rat retina. Investig. Ophthalmol. Vis. Sci..

[24] Lagreze WA, Darstein M, Feuerstein TJ, Otto T, Landwehrmeyer GB (2000). N-methyl-D-aspartate receptor subunit mRNA expression in human retinal ganglion cells. Graefes Arch. Clin. Exp. Ophthalmol..

[25] Yamada,C., Robinson, K., St. Amand, A., Strezoska, Z., Wardle, G., & Anastasia Khvorova, D. L. Innovative technology that enables RNAi in difficult to transfect cells. https://horizondiscovery.com/-/media/Files/Horizon/resources/Posters/accell-difficult-to-transfect-cells-poster.pdf.

[26] Weber C (2012). Toll-like receptor (TLR) 3 immune modulation by unformulated small interfering RNA or DNA and the role of CD14 (in TLR-mediated effects). Immunology.

[27] Yang H (2010). The role of CTGF in the diabetic rat retina and its relationship with VEGF and TGF-β2, elucidated by treatment with CTGFsiRNA. Acta Ophthalmol..

[28] Winkler JL, Kedees MH, Guz Y, Teitelman G (2012). Inhibition of connective tissue growth factor by small interfering ribonucleic acid prevents increase in extracellular matrix molecules in a rodent model of diabetic retinopathy. Mol. Vis..

[29] Kim B (2004). Inhibition of ocular angiogenesis by siRNA targeting vascular endothelial growth factor pathway genes: therapeutic strategy for herpetic stromal keratitis. Am. J. Pathol..

[30] Gorbatyuk M, Justilien V, Liu J, Hauswirth WW, Lewin AS (2007). Suppression of mouse rhodopsin expression in vivo by AAV mediated siRNA delivery. Vis. Res..

[31] Köhr G (2006). NMDA receptor function: subunit composition versus spatial distribution. Cell Tissue Res..

[32] Kageyama M, Ota T, Sasaoka M, Katsuta O, Shinomiya K (2019). Chemical proteasome inhibition as a novel animal model of inner retinal degeneration in rats. PLoS ONE.

[33] Fuwa M (2019). Nafamostat and sepimostat identified as novel neuroprotective agents via NR2B N-methyl-D-aspartate receptor antagonism using a rat retinal excitotoxicity model. Sci. Rep..

[34] Taniguchi T, Shimazawa M, Sasaoka M, Shimazaki A, Hara H (2006). Endothelin-1 impairs retrograde axonal transport and leads to axonal injury in rat optic nerve. Curr. Neurovasc. Res..

[35] Manabe SI, Lipton SA (2003). Divergent NMDA signals leading to proapoptotic and antiapoptotic pathways in the rat retina. Investig. Ophthalmol. Vis. Sci..

[36] Salinas-Navarro M (2009). Retinal ganglion cell population in adult albino and pigmented mice: a computerized analysis of the entire population and its spatial distribution. Vis. Res..

