# Supplementary material for: Novel use of a chemically modified siRNA for robust and sustainable in vivo gene silencing in the retina
Source: Sci Rep. 2020 Dec 18;10:22343. doi: 10.1038/s41598-020-79242-w (PMC7749170; doi:10.1038/s41598-020-79242-w)
Supplement: Supplementary file 1 — Supplementary Information. [file 41598_2020_79242_MOESM1_ESM.pdf]

Novel use of a chemically modified siRNA for robust and sustainable *in vivo* gene silencing in the retina

Takazumi Taniguchi[1*], Ken-ichi Endo[1], Hidetoshi Tanioka[1], Masaaki Sasaoka[2], Kei Tashiro[3], Shigeru Kinoshita[4], Masaaki Kageyama[2]

[1]*Research and Development Division, Santen Pharmaceutical Co., Ltd., Nara, Japan.*

[2]*Global Alliances and External Research, Santen Pharmaceutical Co., Ltd., Nara, Japan.*

[3]*Department of Genomic Medical Sciences, Kyoto Prefectural University of Medicine, Kyoto, Japan.*

[4]*Department of Frontier Medical Science and Technology for Ophthalmology, Kyoto Prefectural University of Medicine, Kyoto, Japan.*

Corresponding author; Takazumi Taniguchi

R&D Division, Santen Pharmaceutical Co., Ltd., 8916-16 Takayama-cho, Ikoma-shi, Nara 630-0101, Tel. +81-743-79-4584 Fax. +81-743-79-4591

E-mail: takazumi.taniguchi@santen.com

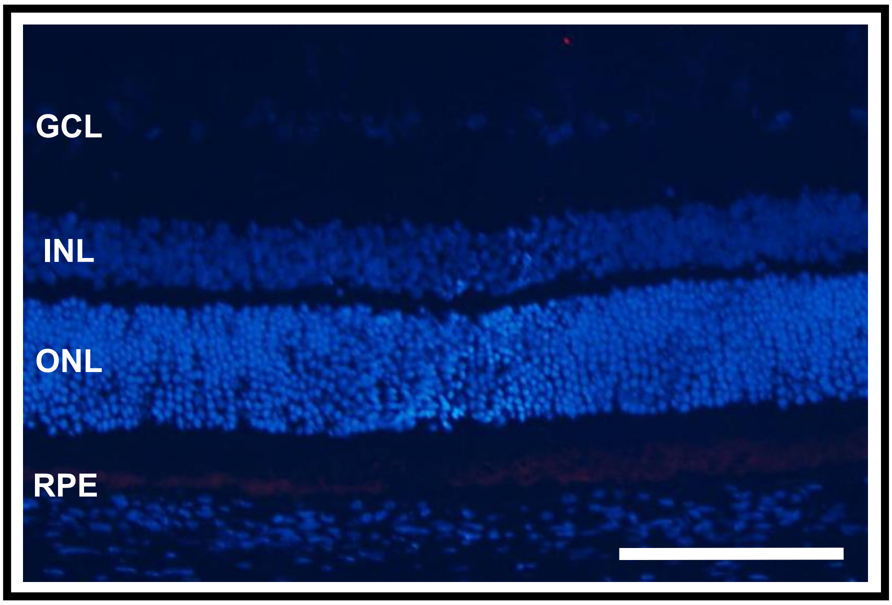

Supplementary Figure S1. A fluorescence microscopic image of the rat retina after intravitreal injection of Dulbecco's phosphate-buffered saline (D-PBS).
Histological analysis of a retinal tissue was performed 3 days after intravitreal injection of D-PBS. The section with DAPI staining was observed under a fluorescence microscope. The scale bar represents 100 µm.
GCL, ganglion cell layer; INL, inner nuclear layer; ONL, outer nuclear layer; RPE, retinal pigment epithelium

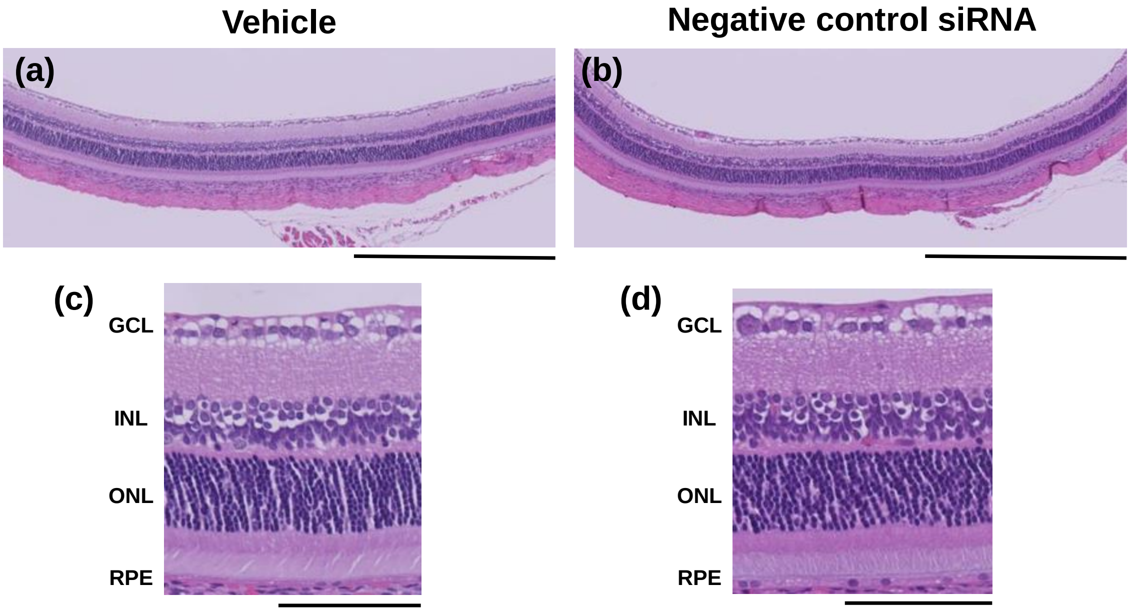

Supplementary Figure S2. Representative images of the rat retina after intravitreal injection of D-PBS or negative control siRNA.

Histological analysis of retinal tissues was performed 7 days after intravitreal injection of vehicle (D-PBS) or negative control siRNA (7.0 nmol/eye). Retinal cross-sections were prepared and stained with hematoxylin and eosin. (a) Vehicle and (b) negative control siRNA in lower magnification (the scale bars represent 500 µm); (c) Vehicle and (d) negative control siRNA in higher magnification (the scale bars represent 100 µm).

GCL, ganglion cell layer; INL, inner nuclear layer; ONL, outer nuclear layer; RPE, retinal pigment epithelium
